# Pre-Anthesis Cytokinin Applications Increase Table Grape Berry Firmness by Modulating Cell Wall Polysaccharides

**DOI:** 10.3390/plants10122642

**Published:** 2021-12-01

**Authors:** Bárbara Rojas, Felipe Suárez-Vega, Susana Saez-Aguayo, Patricio Olmedo, Baltasar Zepeda, Joaquín Delgado-Rioseco, Bruno G. Defilippi, Romina Pedreschi, Claudio Meneses, Alonso G. Pérez-Donoso, Reinaldo Campos-Vargas

**Affiliations:** 1 Centro de Estudios Postcosecha, Facultad de Ciencias Agronómicas, Universidad de Chile, Santiago 8831314, Chile; biobarbara.rsm@gmail.com (B.R.); pa.olmedo@gmail.com (P.O.); 2 Centro de Biotecnología Vegetal, Facultad de Ciencias de la Vida, Universidad Andrés Bello, Santiago 8370186, Chile; susana.saez@unab.cl (S.S.-A.); joaquin.delgado.rioseco@gmail.com (J.D.-R.); claudio.meneses@unab.cl (C.M.); 3 Departamento de Fruticultura y Enología, Facultad de Agronomía e Ingeniería Forestal, Pontificia Universidad Católica de Chile, Santiago 7820436, Chile; fgsuarez@uc.cl; 4 Horticulture and Product Physiology, Department of Plant Sciences, Wageningen University, P.O. Box 16, 6700 AA Wageningen, The Netherlands; baltasar.zepedapuiggros@wur.nl; 5 INIA La Platina, Instituto de Investigaciones Agropecuarias, Santiago 8831314, Chile; bdefilip@inia.cl; 6 Escuela de Agronomía, Facultad de Ciencias Agronómicas y de los Alimentos, Pontificia Universidad Católica de Valparaíso, Quillota 2260000, Chile; romina.pedreschi@pucv.cl

**Keywords:** *Vitis vinifera*, pre-anthesis, cytokinin, CPPU, cell wall, Thompson Seedless

## Abstract

The use of plant growth regulators (PGRs) is widespread in commercial table grape vineyards. The synthetic cytokinin CPPU is a PGR that is extensively used to obtain higher quality grapes. However, the effect of CPPU on berry firmness is not clear. The current study investigated the effects of pre-anthesis applications (BBCH15 and BBCH55 stages) of CPPU on ‘Thompson Seedless’ berry firmness at harvest through a combination of cytological, morphological, and biochemical analyses. Ovaries in CPPU-treated plants presented morphological changes related to cell division and cell wall modification at the anthesis stage (BBCH65). Moreover, immunofluorescence analysis with monoclonal antibodies 2F4 and LM15 against pectin and xyloglucan demonstrated that CPPU treatment resulted in cell wall modifications at anthesis. These early changes have major repercussions regarding the hemicellulose and pectin cell wall composition of mature fruits, and are associated with increased calcium content and a higher berry firmness at harvest.

## 1. Introduction

Grapevine (*Vitis vinifera* L.) is among the most valuable crops, with 80 million tons produced globally every year [1]. Several traits are commercially significant for table grapes such as berry size, sugar content, and firmness [2,3,4]. Thus, this crop has been accompanied by the use of plant growth regulators (PGRs) to sustain economically viable production and increase its commercial value [5]. Among PGRs, synthetic vegetal hormones are the most frequently used, with gibberellic acid (GA_3_), synthetic cytokinins such as 6-benzyl aminopurine (6-BA) and thidiazuron (TDZ), and the urea-derivate synthetic cytokinin N-(2-chloro-4-Pyridyl)-N-Phenyl urea (CPPU, forchlorfenuron) [5,6] all being used. However, the combination of two or more PGRs at different stages of grape berry development is an extended agronomical practice [3,6].

The use of different PGRs in terms of chemical nature and application timing is accompanied by the knowledge of developmental and growing phases of *Vitis vinifera* flowers and fruits to optimize grape berry quality. Grape flowers are arranged in a raceme-type known as an inflorescence, with a protective cap covering the reproductive organs, five stamens, and a surrounding single ovary with two locules and four ovules [4]. During anthesis, the cap detaches from the base, and the ovary begins developing into a fleshy fruit to become the pericarp of the berry [4,7]. According to morphogenesis studies, the ovary wall starts to grow in the first phase through an active division of mesocarp cells, gradually changing to cell enlargement, followed by a lag phase and continuing with the third phase in which cell enlargement occurs and ripening begins [8]. For grape stenospermocarpic cultivars—which correspond to a seedless fruit, such as Thompson Seedless—the ovules are fertilized, but the embryos abort early and do not develop seeds [9]. The biological processes mentioned above are accompanied by endogenous hormonal changes, including high levels of gibberellic acid and cytokinin at early stages of fruit development that drive a rapid cell division and enlargement in grape berries [10], whose function is to carry out fruit set and the early growth, followed by tissue differentiation and fruit softening as a result of ripening when lower hormonal levels are reached [11].

Despite increased berry size and delayed senescence being described through cytokinin (CPPU) applications [12], there is a lack of information regarding the effect of CPPU treatments on grape berry firmness. Firmness is a complex attribute comprising the mechanical and sensorial parameters of a fruit, and the instrumental measurements of firmness have been described as correlating with sensory panel descriptions and consumer acceptability [13]. It has been reported that CPPU-treated plants produce firmer fruit, such as apples, where linearly increasing fruit firmness was observed with increasing CPPU concentrations [14] and a higher fruit firmness was described in cucumber after CPPU applications [15]. For the hybrid *V. labrusca* × *V. vinifera* ‘Himrod’, post-flowering CPPU applications induced increased fruit firmness [10]; however, pre-anthesis CPPU treatments have been poorly studied in *Vitis vinifera*. Interestingly, a recent study reported an increase in ovary and grape berry size by applying CPPU at the early stages of floral development (BBCH15 and BBCH55; [16]) on Thompson Seedless cultivar when inflorescences were clearly defined [17]. Since a high rate of cell division has been described before anthesis compared to after anthesis [7], the application of PGRs before flowering appears to be critical for inducing changes in fruit ontogeny and molecular processes, including cell wall dynamics.

A previous study indicated that cytokinin are involved in controlling the balance between cell division and differentiation [18], followed by a complex remodeling of the plant cell wall architecture [19]. The plant cell wall is a highly dynamic structure that surrounds the plant cell membrane influencing plant development, cell morphology, cell division, and also acts as the first physical barrier between the inside and the outside of the plant cell [20]. The primary cell wall is mainly composed of cellulose, hemicellulose, and pectin, forming a complex network that can be modified and restructured to allow cell wall expansion required during morphological cell changes, including cell division and cell differentiation [21]. Cellulose and hemicelluloses are mostly built of neutral sugars that are mainly bonded by β-(1,4) linkages that provide the tensile strength of the cell wall, while pectin forms a soft tensile gelatinous network implicated in cell wall porosity and cell–cell adhesion [22]. Pectin polysaccharides are characterized by their high acidic sugar content, and are mainly composed of galacturonic acid (GalA) residues connected by α-(1,4) linkages forming four domains characterized as homogalacturonan (HG), xylogalacturonan (XGA), rhamnogalacturonan-I (RG-I) [23], and rhamnogalacturonan-II (RG-II) [24]. HG, XGA, and RG-II are formed by a backbone of GalA residues, which can be unbranched (HG), substituted with xylose (Xyl) residues (XGA), or decorated with a conserved set of complex side chains [25]. RG-I differs from these three pectin domains because its backbone is formed by a repeated α-D-(1,4)-GalA-α-L-(1,2)-Rha (rhamnose) disaccharide that can be substituted with side-chain structures containing both galactose (Gal) and arabinose (Ara) units [26]. Additionally, a recent study has described the implications of cell wall composition in berry fruit size and firmness, suggesting the relevance of cell wall polysaccharide architecture in this complex process and producing interesting commercial and agronomical traits [27].

In the present work, we aimed to determine the effect of CPPU applications during the early stages of pre-anthesis and its impact on grape berry firmness at harvest through changes in cell wall dynamics.

## 2. Results

### 2.1. Cell Wall and Cell Cycle-Related Gene Expression Analysis in Response to CPPU Applications by RT-qPCR

**Figure 1:**
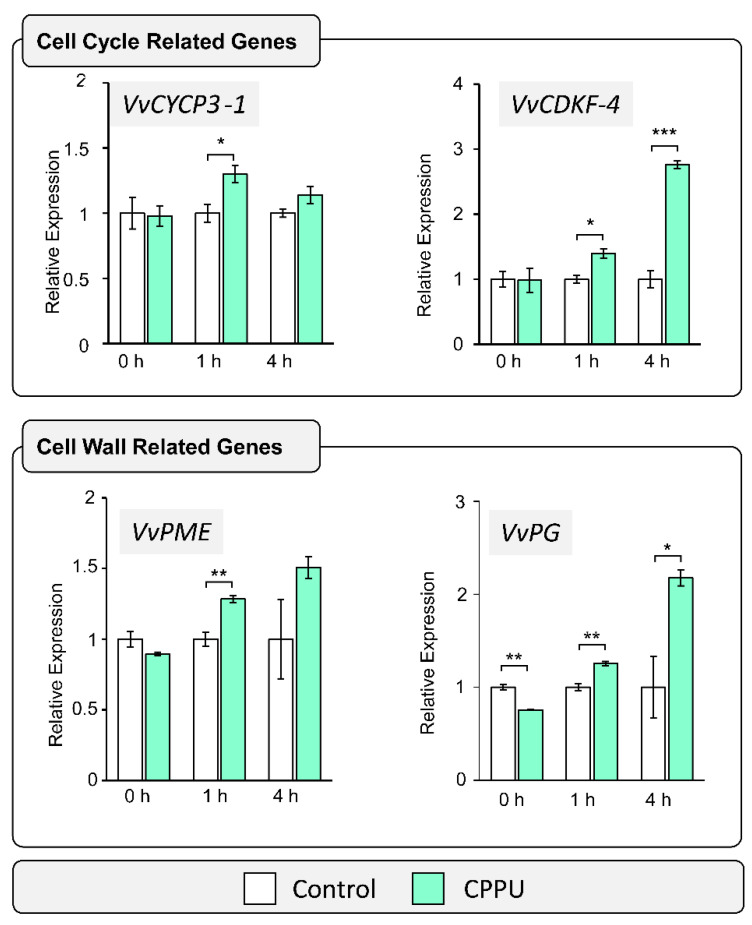
Early response of cell wall and cytokinin-related genes to CPPU treatment. Transcript relative abundance was determined by RT-qPCR at 0, 1, and 4 h since CPPU was applied to inflorescences at BBCH15 stage. Two cell cycle-related genes were evaluated: a cyclin (*VvCYCP3-1*; GSVIVG01011079001) and a cyclin-dependent kinase (*VvCDKF-4*; GSVIVG01022771001). Additionally, the transcript levels of a pectin methylesterase (*VvPME*; GSVIVG01028041001) and a polygalacturonase (*VvPG*; GSVIVG01026985001) were analyzed. Expression levels were normalized to the transcript levels of the housekeeping gene transcriptional elongation factor II (*VvEFIα*; GSVIVG01025147001). Results show mean ± SE from *n* = 3, and asterisks indicate statistical significance as compared to control condition and determined by Student’s *t*-test (*, *p* ≤ 0.05; **, *p* ≤ 0.01; ***, *p* ≤ 0.001). An independent test was performed at each time point.

Phytohormones such as cytokinins have been described as key factors controlling the equilibrium between cell division, cell differentiation, and cell wall reorganization [28]. To assess their immediate CPPU effect on treated inflorescences, cell cycle and cell wall specific transcript abundances at the pre-anthesis BBCH15 stage were examined by RT-qPCR (Figure 1). A previous work showed that CPPU applications at early stages of inflorescence development prevented postharvest disorders [17], suggesting that the BBCH15 growth stage—where inflorescence is clear and displays five separate leaves—and the BBCH55 growth stage—where flowers are in compact groups and display eight separate leaves—are key stages for CPPU application. Cell cycle-related genes *VvCYCP3-1* (cyclin-U-3-1; GSVIVG01011079001) and *VvCDKF-4* (cyclin-dependent kinase-F-4; GSVIVG01022771001) were selected for analysis according to early stage expression criteria and the functional annotation using the GENOSCOPE database (https://www.genoscope.cns.fr/externe/GenomeBrowser/Vitis/, accessed on 22 November 2021) from a transcriptomic analysis previously described [29], where both genes were identified as key cell cycle regulators in response to cytokinin [30]. Relative transcript levels of both cell cycle-related genes were evaluated at 0, 1, and 4 h after CPPU application in grape inflorescences. Both *VvCDKF-4* and *VvCYCP3-1* genes were expressed constitutively between 0 and 4 h, showing a stable level in control inflorescences (Figure 1). For treated samples, a significant increase was observed in the *VvCYCP3-1* gene expression 1 h after CPPU application, followed by a decrease in basal accumulation level detected 4 h after CPPU treatment. On the other hand, the *VvCDKF-4* gene showed a different expression pattern after CPPU application by increasing the transcript accumulation at 1 and 4 h, suggesting that the phytohormone influenced cell cycle regulation and progression. Due to cell division requiring synchronization with cell wall biosynthesis and reorganization [19], changes in cell wall-related gene expression were analyzed. Since pectin is the most abundant component of grape berry cell walls [31], two enzymes involved in HG modifications were studied. The transcript levels of *VvPME* (Pectin methylesterase; GSVIVG01028041001) and *VvPG* (Polygalacturonase; GSVIVG01026985001) were evaluated at 0, 1, and 4 h after CPPU application. Data showed a significant accumulation of the *VvPME* transcript after 1 h of CPPU treatment. Additionally, *VvPG* exhibited a significant decrease at 0 h, suggesting a variation in *VvPG* transcript levels between individual inflorescences collected. However, a significant increase in transcript accumulation of *VvPG* was detected at 1 h and 4 h after CPPU application. These findings support the hypothesis that an increased cell division mediated by CPPU applications involves a cell wall restructuration to allow morphological changes.

### 2.2. Cell Count and Inflorescence Size Analysis at Pre-Anthesis Stage

**Figure 2:**
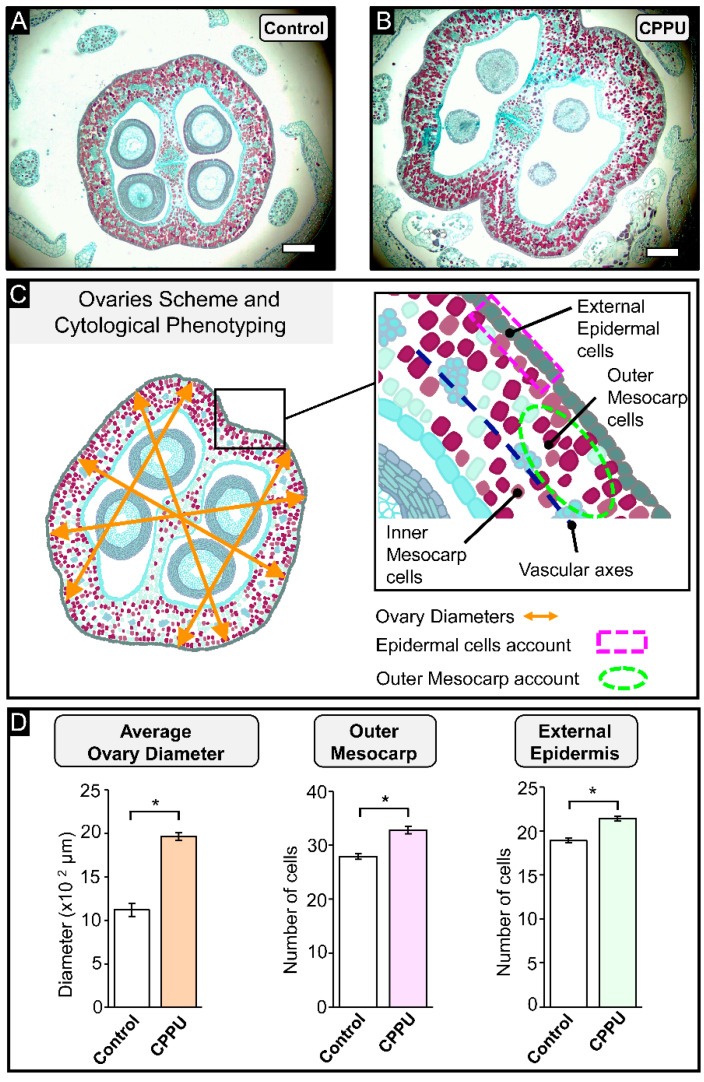
Pre-anthesis CPPU treatment modifies the ovary morphology at the anthesis stage: cross-section of cv. Thompson Seedless untreated (**A**) with two CPPU applications (**B**). Schematic representation of measurements on grape ovary images using Fiji software (**C**): the average ovary diameter was calculated with five measures across the septum and locules (*n* = 30). The number of cells in the external epidermis (EE) was established by determining a 200 μm straight line and counting the number of cells in that section (*n* = 30). The number of cells in the outer mesocarp (OM) was calculated by taking a 5000 μm² area and determining the number of cells in that area (*n* = 30). In (**D**), the results show mean ± SE, and asterisks indicate statistical significance as compared to control condition and determined by Student’s *t*-test (*, *p* ≤ 0.05). Bar = 100 μm.

To determine the effect of CPPU applications at the grapevine flowering stage, light microscopy was used to evaluate differences among CPPU-treated and control inflorescences at the morphoanatomical level using safranin and Fast Green staining, which stains nuclei, nucleoli, and lignin accumulation in red, and cellulose walls and cytoplasm in green, respectively (Figure 2A,B). The cells of the first tissue zone are stained cyan/green color, indicating cell wall and cytoplasm, while the outer mesocarp cells are stained red by the presence of high phenolic content [32]. The resultant bright-field microscopy images show enlarged ovaries of inflorescences treated with CPPU compared to control inflorescences (Figure 2A,B), evidencing the strong effect of this synthetic phytohormone on grapes inflorescences. An ovary scheme is shown in Figure 2C to explain the different parameters measured to assess the ovary morphological changes at the flowering stage after CPPU applications. Two tissue organization zones can be clearly distinguished in equatorial cross-sections of ovary walls delineated by a circle of 25 to 40 vascular bundles, i.e., the inner and outer mesocarp (Figure 2C). Three parameters were determined: average ovary diameter, number of cells from the outer mesocarp (per a given surface area, i.e., mesocarp cell density), and number of cells from the external epidermis (per a given arc, i.e., epidermal cell density) (Figure 2D). An increase of 54.2% in the average ovary diameter of the inflorescence was registered in CPPU-treated inflorescences compared to control inflorescences (Figure 2D, orange bar). CPPU-treated inflorescences showed significant increases of 17.7% and 13.2% in the cell number from the outer mesocarp and from external epidermis compared to control inflorescences, respectively (Figure 2D, pink bar and light green bar). These observations suggest that early CPPU treatments at BBCH15 and BBCH55 stages (pre-anthesis) trigger an increased cell division, as evidenced by a larger ovary size (diameter) and higher tissue cell density at the outer mesocarp and epidermis, implying a larger total number of cells in the cross-section and perimeter of CPPU-treated ovaries.

### 2.3. Immunohistochemical Analysis of CELL Wall Structure from CPPU-Treated Inflorescences

**Figure 3:**
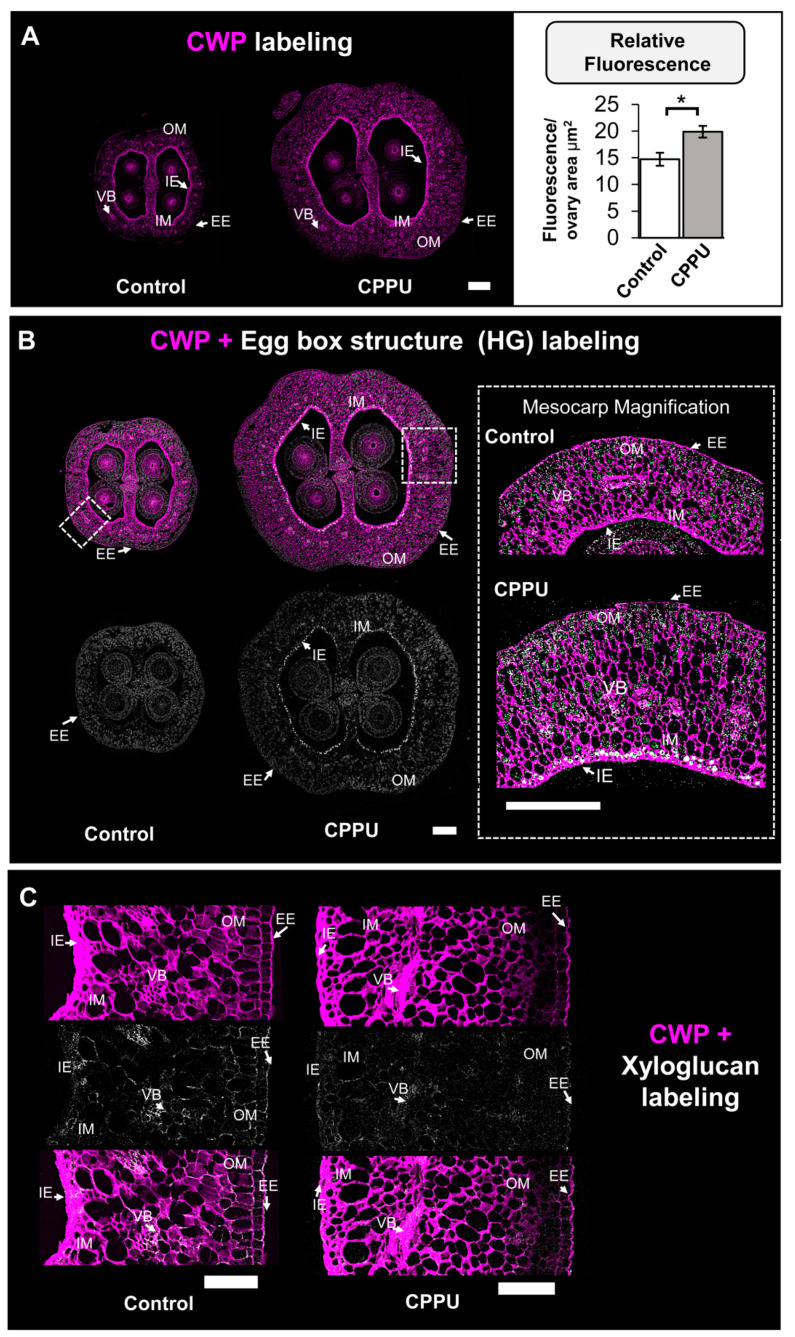
Pre-anthesis CPPU applications induce changes in ovary cell wall content at flowering stage. Immunolabeling of cross-sections from control and CPPU-treated ovaries: (**A**) Calcofluor White was applied to stain cell wall polysaccharides (CWP) (magenta), and a representative cross-section of each ovary is shown; scale bar: 200 µm. The right figure exhibits the relative fluorescence signal of Calcofluor White as normalized values regarding total ovary area, and the results show mean ± SE from five biological replicates (*n* = 5); asterisks indicate statistical significance as compared to control condition and determined by Student’s *t*-test (*, *p* ≤ 0.05). (**B**) Cross-sections of ovaries were immunolabeled with 2F4 monoclonal antibody (gray) to target the egg-box structure. Scale bar: 200 μm; a mesocarp magnification is displayed to evidence high signal in the internal epidermis (IE). IM = internal mesocarp; VB = vascular bundle; EM = external mesocarp; EE = external epidermis; OM = outer mesocarp. (**C**) Representative sections of the ovary wall immunolabeled with Calcofluor White (magenta) and LM15 monoclonal antibody (gray) to target the XXXG motif of xyloglucan. Images of ovary were digitally extracted and placed on the same background for phenotype comparison. Scale bars = 200 μm.

**Figure 4:**
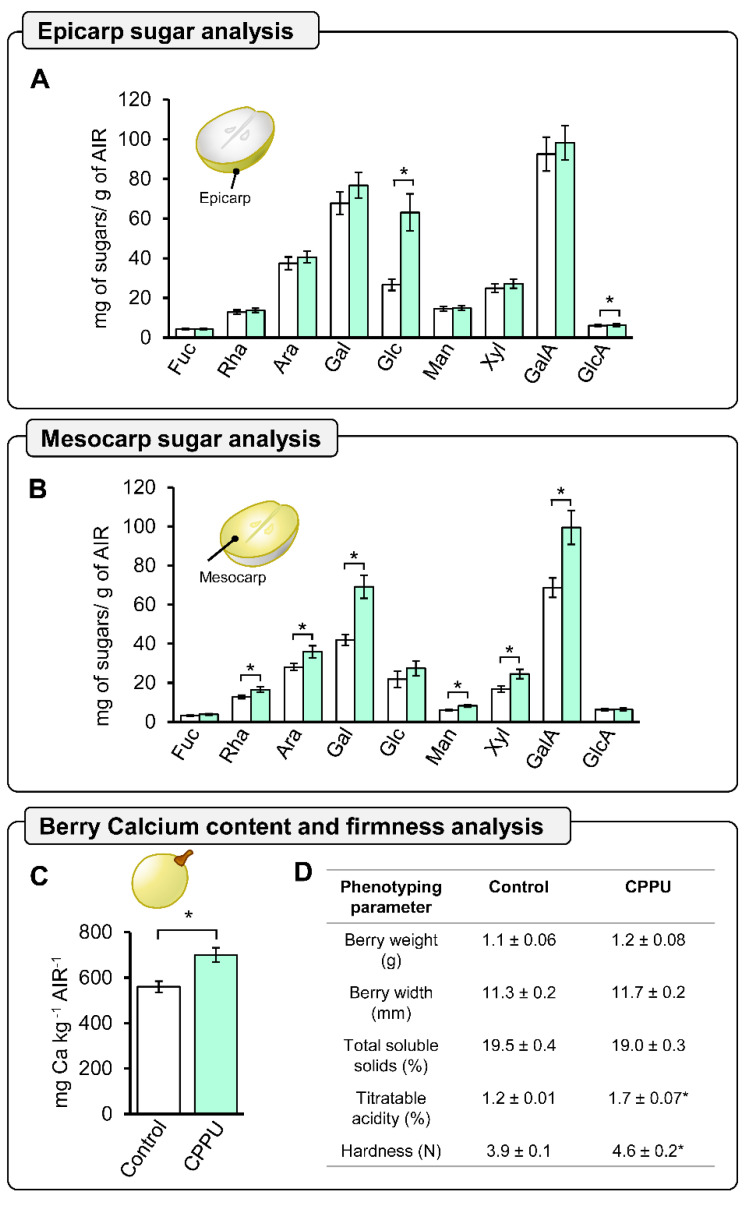
CPPU treatment increases berry firmness associated with changes in sugar cell wall composition and a higher accumulation of intracellular calcium. Sugars in cell wall alcohol insoluble residue (AIR) from the epicarp (**A**) and mesocarp (**B**) berries were quantified by high-performance anion-exchange chromatography with pulsed amperometric detection (HPAEC-PAD). Fuc = fucose; Rha = rhamnose; Ara = arabinose; Gal = galactose; Glc = glucose; Man = mannose; Xyl = xylose; GalA = acid galacturonic; GlcA = acid glucuronic. (**C**) The calcium content of the AIR was determined by inductively coupled plasma optical emission spectrometry (ICP-OES), and three biological replicates are shown (*n* = 3). (**D**) The table with the main phenotyping parameters is displayed with hardness as firmness (*n* = 25). The results show mean ± SE, and asterisks indicate statistical significance compared to control condition and determined by Student’s *t*-test (*, *p* ≤ 0.05).

Based on the differences in ovary size and cell number shown above, the comparison of the cell wall content of the control and CPPU-treated inflorescences could provide valuable information on the cell adjustments induced by CPPU applications. To investigate changes in ovary cell wall content, the structure and distribution of HG epitopes in ovary cell walls were examined by immunolabeling on cross-sections. The images obtained from confocal microscopy showed an increase in ovary size previously observed with cytological analysis. In addition to the increased size observed in CPPU-treated inflorescences, the ovary exhibited more cell wall polysaccharides fluorescent signal than the control inflorescences in the outer mesocarp from ovary cells (Figure 3A, left panel). Fluorescence quantifications support these results, indicating a significant increase of 34.7% in cell wall polysaccharides labeling from the CPPU treatment compared to the control (Figure 3A, right panel). The most abundant grape berry cell wall polysaccharides are HGs (from pectins) and XyGs (from hemicelluloses) [31,33]. To evaluate whether these relevant molecule structures are modified in the ovary cell walls of CPPU-treated samples, immunohistochemical assays were performed on cross-sections using 2F4 and LM15 antibodies raised against an ‘egg-box’ motif—a dimer structure formed by crosslinked homogalacturonans and calcium ions—and xyloglucan (XyG), respectively [34,35]. In control ovaries, a 2F4 signal was detected within all the mesocarp cells with a regular signal (Figure 3B). CPPU-treated ovaries also present a labeling in all mesocarp cells, but, in contrast with the control, the inner epidermis cell layer exhibits a stronger labeling (Figure 3B, mesocarp magnification). For xyloglucan epitopes labelled by LM15 antibody, a higher signal intensity in the epidermal cells of the inner and outer mesocarp, and vascular cells, was observed (Figure 3C). The CPPU-treated ovary exhibits less labeling within peripherical cells and vascular bundles in comparison to the control (Figure 4C), suggesting a differential XyG accumulation in grape berry tissues. The data obtained from confocal microscopy suggest a different cell wall content that could impact grape berry firmness at later ripening stages.

### 2.4. Global Cell Wall Non-Cellulosic Monosaccharide Composition Analysis of CPPU-Treated Grape Berries at Harvest by HPAEC-PAD

To elucidate whether the changes observed at anthesis stage were coherent with grape berries at harvest, cell wall monosaccharide composition analyses of cell wall material derived from epicarp and mesocarp tissues were conducted separately. Analyses obtained from epicarp tissue showed significant differences for glucose and glucuronic acid, which were higher in CPPU-treated grape berries than in control (Figure 4A). However, for the mesocarp tissue, rhamnose, arabinose, galactose, mannose, xylose, and galacturonic acid were detected in significantly higher concentrations in CPPU-treated grape berries in comparison to the control (Figure 4B). GalA content in CPPU-treated grape berry mesocarp is 31% higher compared to control grape berries. Additionally, xylose and galactose residues, which are both involved in XyG composition, showed significantly higher accumulations of 45.6% and 64.8% compared to control grape berry mesocarp (Figure 4B), respectively. Since calcium has been associated with cell wall material in the participation in the formation of HG dimers egg-box, we analyzed the calcium concentration present in the cell wall material (alcohol-insoluble residue, AIRs) obtained from CPPU-treated and control grape berries at harvest. Interestingly, CPPU-treated grape berries exhibited a significantly increased quantification of cell wall-associated calcium, which was 29.4% higher compared to the control (Figure 4C), suggesting that CPPU applications could play an important role in the formation of cell wall egg-box motifs.

### 2.5. Phenotype and Firmness Properties of CPPU-Treated Table Grapes

To obtain a better understanding of the relation between changes in cell wall composition and phenotypic features, a characterization of the berry weight, berry width, total soluble solids, titratable acidity, and hardness of CPPU-treated and control grape berries was performed at harvest. Texture profile analysis (TPA) is composed of several parameters, with hardness being considered a suitable indicator of firmness in different berries [27,36]. The CPPU-treated and control grape berries exhibited similar weights, widths, and total soluble solids, but contrasting titratable acidity and hardness (firmness) (Figure 4D). The CPPU-treated grape berries showed a significantly increased firmness, which was 18% higher than the control grape berries, suggesting that CPPU treatment could induce cell wall modulation associated with fruit firmness (Figure 4D).

## 3. Discussion

This work reveals that pre-anthesis CPPU applications in grape inflorescences modulate changes in cell wall composition and distribution of floral tissues, which correlate with an increased grape berry firmness at harvest. These findings expand the scope of the evidence in support of the hypothesis that cytokinins play an important role in cell wall metabolism and cell wall calcium associated with grape berry texture.

### 3.1. Cell Wall and Cell Cycle-Related Transcript Expressions Are Differentially Accumulated in Response to Early CPPU Applications

It is understood that cytokinins, as cell division promoters, control checkpoints and promote transitions at G2/M and G1/S in which CDKs participate, supporting the observation of increased cell number and size when phytohormone is exogenously administered [30,37]. As many cyclins can be specific to the organ or development stage, the genes selected for this work were chosen based on a fruit transcriptome [29]. The data obtained contributed to the idea that CPPU induced morphological changes in cells, as displayed in the ovary sections. Previous studies have reported a response to cytokinins at the transcript level, and gene cyclin expression is rate-limiting for cell division [37,38]. Related to cell wall-related genes and among pectin polysaccharides, the enriched GalA HG domain is a key molecule, since its modifications, particularly its degree of methylesterification, have a direct relation to the biomechanical properties of the cell wall [21]. HG polymers are synthesized by diverse galacturonosyltransferases and are methylesterified in the Golgi apparatus before their secretion into the apoplast [24]. Once HGs are deposited in the apoplast, their methylesterification status is regulated by specific HG-modifying enzymes known as pectin methylesterases (PMEs), which catalyze HG demethylesterification [21]. HG demethylesterification in the cell wall can have different consequences: (1) the formation of the so-called egg-box structures by Ca^2+^-crosslinking between demethylesterified HG chains, which contributes to cell wall stiffening [34], or (2) its catabolism by HG-degrading enzymes such as polygalacturonases or pectate lyases (PG/PL), which promote cell wall loosening by the relaxation of the pectin network [21]. In the grapevine fruit model, *VvPME* and *VvPG* are codified by 47 and 36 genes, respectively, and the expression patterns are different in developmental stages and tissues [39]. The role of cytokinins in cell wall-related genes is poorly understood, and the data suggest that CPPU applications induce an increase in cell wall-associated gene expression; however, it has been also been noted that the inhibition of cell wall biosynthesis promotes cytokinin degradation and thus decreases cell division [40]. These data open new perspectives on cell wall-related gene expression modulation by CPPU applications.

### 3.2. CPPU Treatment Increased Cell Count and Inflorescence Size at Anthesis Stage

Grape berry fruit development is characterized by four stages: ovary development, fruit set, expansive fruit growth, and maturation/ripening [7,41]; anthesis or flowering (BBCH65) is timed between ovary development and fruit set immediately before fertilization. The substantial changes in ovary diameter and tissue cell density suggest that CPPU treatment could certainly influence ovary size, and these observations correlate with exogenous cytokinin application at post-anthesis, increasing berry size [12]; recently, the same effect was reported with a pre-anthesis CPPU application in grapes [17]. Evidence that cell division and enlargement of ovary cells are the main factors responsible for the final size of fleshy fruits has been reported [8,41]. These crucial morphological cell changes take place before anthesis and are easily visible at this stage [42]. Thus, the flowering or anthesis stages are important stages in which to evaluate cell number and ovary size in this study. The morphological changes in the ovary are an excellent parameter that must be analyzed during ovary ontogenesis; the peripheral ovary wall, composed of two single-cell-layered epidermises and the mesocarp parenchymal-like tissue, represents the significant part of the berry where the outer mesocarp gives origin to the epicarp, while the inner mesocarp generates fruit flesh [4,7,43]. Moreover, cell division is one of the decisive factors in fruit organogenesis, supporting the role of the phytohormone CPPU in determining traits such as berry size, firmness, and biochemical composition in the early stages of development [42].

### 3.3. CPPU Treatment Induces Changes in Ovary Cell Wall Content

The results presented using an optical approach suggest that an increased number of cells in the ovary is potentially accompanied by changes in cell wall composition and cell epitope redistribution associated with cell wall content in the CPPU-treated samples. During later stages in development, the inner and outer mesocarp cells are differentiated in the flesh and epicarp fruit tissues, respectively. Recent studies in blueberries’ epicarp and mesocarp tissues have described that the egg-box motif, formed by cross-linking between demethylesterified HG and calcium, and xyloglucan polymers are key molecules involved in fruit firmness and fruit size determination [27,44]. Thus, the increase in 2F4 labeling implies the presence of an egg-box structure in a group of cells, suggesting a stiffening of these cell layers that form the future mesocarp [4]. Such differences are likely indicative of the early effect of CPPU on inflorescences at the cell wall level, mainly in the genesis of firmer berries, due to calcium bridges correlating with higher cell wall stiffness in grape berries [2]. The differences in the xyloglucan epitope distribution suggest modifications in their structure but we cannot exclude the fact that they could be masked by the other polymer components or changes in the density of the cell wall polysaccharide network [35,45]. The immunohistochemical differences between control and CPPU-treated inflorescences at anthesis propose an early pectin remodeling in response to the exogenous CPPU phytohormone. During fruit development, the composition and polysaccharide structure continuously changes, and it is possible at this stage to evidence future cell wall changes derived by CPPU applications at full bloom [21,24].

### 3.4. Early CPPU Treatment Changes Cell Wall Composition and Promotes an Increase in Berry Firmness at Harvest

The cell wall is a complex matrix structured by three main components: cellulose, hemicellulose, and pectin [20,46]. The increase in glucose content from CPPU-treated epicarp tissue could be correlated with the strong labeling of calcofluor to cellulose and hemicellulose in the cell wall of CPPU-treated ovaries [47,48], supporting the notion that CPPU applications induce changes that increase cellulose deposition and hemicellulose trafficking in the epicarp tissue from grape berries. However, the glucose amount detected by 2 M trifluoroacetic acid (TFA) hydrolyzation corresponds mainly to non-cellulosic monosaccharides, suggesting hemicellulose dynamics [49]. Regarding hemicellulose, epicarp and mesocarp tissues contain an increased amount of different XyG monosaccharides, suggesting that pre-anthesis CPPU applications could promote xyloglucan synthesis during grape berry development. Homogalacturonan is the pectinic domain of cell wall components most widely associated with firmness in different berry fruits [2,27,50,51,52]. The analyses indicate that pre-anthesis CPPU applications induce an increased accumulation of GalA and cell wall-associated calcium than control samples in the mesocarp tissue. These findings correlate with the presence of the egg-box structure observed in the inner mesocarp cells at anthesis, suggesting that the presence of this dimerization could prevent HG degradation by HG-degrading enzymes, such as polygalacturonases and pectate lyases [53]. Furthermore, a previous study described a correlation between an increase in the calcium content of berries and higher firmness concomitant with the role of this cation in the HG egg-box structure [27]. The analysis of cell wall composition indicates that pre-anthesis CPPU applications have an impact in the mesocarp tissue, exhibiting an overall increased accumulation of sugar monomers in the cell wall and suggesting an increased amount of pectin and hemicellulose in CPPU-treated grape berries. A previous work applied cytokinin 6-benzylaminopurine (6-BA) at the anthesis stage and no differences were observed in mesocarp tissue morphology, suggesting that the timing of cytokinin applications in the floral stages of grapevine is critical for obtaining desired effects on grape berries after anthesis [54]. Previous studies on grapes have reported the correlation between CPPU application and an increased firmness at harvest [3,10,12,17]. During the first growth phase in grape berry development, there is a major cell division process, and the tissue total cell number is established and followed by cell enlargement [7,41]. This is the most suitable stage for applying PGRs in agronomical practices, such as gibberellic acid, to achieve fruit of a higher caliber [12,54]. A few studies have determined the effect of CPPU applications on firmness before anthesis, such as [14,55]. Such changes arise early during flowering and have an impact on berry firmness at harvest. However, it is tempting to speculate that differences in fruit texture are the result of crosstalk of additional phytohormones; thus, further analysis is required to elucidate this hypothesis.

## 4. Materials and Methods

### 4.1. Plant Material and CPPU Treatments

**Figure 5:**
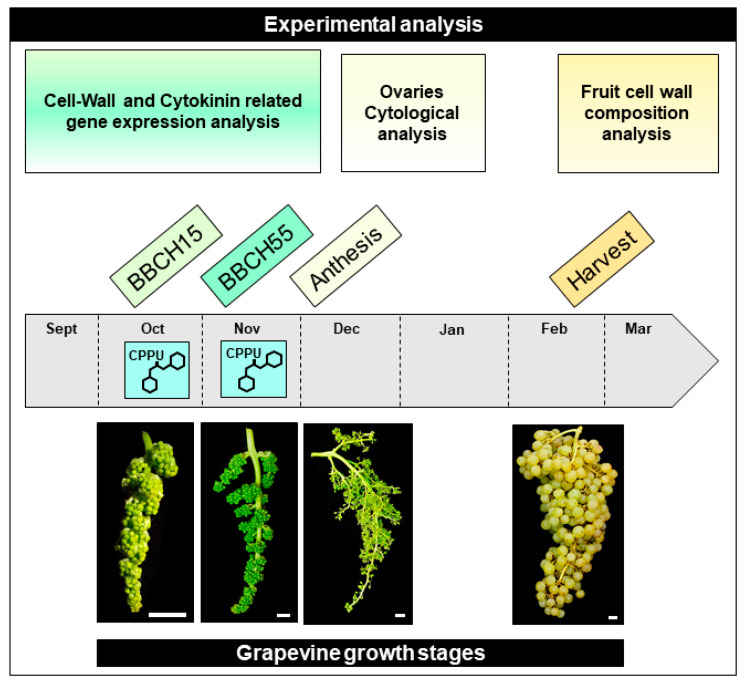
Schematic representation of the experimental design and timeline of grapevine growth stages analyzed in this study. Two CPPU applications were sprayed on inflorescences, the first one at BBCH15 and the second one at the BBCH55 grapevine growth stages. Samples collected at those points were used for relative gene expression analysis targeting cell wall-related and cytokinin-responsive genes. In the same growing season, at the BBCH65 stage (Flowering), when 50% of the caps were off, samples were collected to proceed with the ovary cytological assays by optical and confocal microscopy. In addition, control and treated berries (CPPU) were evaluated for their phenotypes (e.g., hardness, size, and weight) and cell wall composition by HPAEC-PAD, and cell wall calcium content was determined at harvest (BBCH89). Scale Bars = 1 cm.

The trial was conducted during the 2018 and 2019 growing season in a 20-year-old vineyard of cv ‘Thompsons Seedless’ from the Agriculture Experimental Station of Pontificia Universidad Católica de Chile, located at Pirque, Maipo Valley, Chile (33°40′14.8″ S, 70°35′42.5″ W). The CPPU (forchlorfenuron, N-(2-chloro-4-pyridinyl)-N′-phenylurea) treatments were carried out at the BBCH15 growth stage, when inflorescences were clear and displayed five leaves in the shoots, and at the BBCH55 growth stage, when inflorescences were in compact groups and displayed eight separate leaves, of the grapevines (Figure 5) [16], using a 6 mg L^−1^ CPPU–water solution (Agromil Plus^®^, Agroenzymas, Chile). The experimental design was completely randomized, where two consecutive applications of CPPU at 37 DBA (days before anthesis, BBCH15) and then at 26 DBA (BBCH55) were hand-sprayed on inflorescences until they were completely soaked (CPPU plants); as a negative control (control plants), a group of grapevines was sprayed with distilled water. Samples of inflorescences at the BBCH15 stage were collected at 0, 1, and 4 h after the time of CPPU application for control and CPPU plants. Then, they were immediately frozen in liquid nitrogen and stored at −80 °C until RNA extraction and further analysis. Additionally, inflorescences were obtained at anthesis (BBCH65; 0 DBA) and fixed in an FAA solution (3.7% formaldehyde *v*/*v*; 5% glacial acetic acid *v*/*v*; 50% ethanol *v*/*v*) to perform immunohistochemistry and morphology assessments. Mature berries were harvested using commercial ripening stage criteria [16]; thus, CPPU and control grape bunches contained 20 °Brix (% *w*/*w* of g sucrose per 100 g solution) at harvest.

### 4.2. RNA Extraction and cDNA Synthesis

The total RNA was extracted from inflorescences separated from the rachis, using an optimized hot borate method to process myriad samples simultaneously with high quality, as described by [56], with some modifications. Briefly, 100–150 mg of ground tissue was added to 1.5 mL tubes pre-heated to 80 °C extraction buffer (0.2 M sodium borate dehydrate; 30 mM EGTA; 1% SDS *w*/*v*; 1% sodium deoxycholate *w*/*v*; 2% PVP *w*/*v*, 10 mM DTT, 1% NP-40 *v*/*v*; pH 9), and mixed vigorously. After that, it was incubated for 1.5 h with 500 mg/mL proteinase K (Sigma-Aldrich, St. Louis, MO, USA). After overnight LiCl precipitation, the RNA was recovered in a pellet by centrifugation. Successive washes were completed. The RNA precipitation with 100% ethanol was performed for 3 h at −20 °C to continue with centrifugation for 30 min at 12,000× *g* at 4 °C, and washed with 80% EtOH. RNA pellet was dried at room temperature followed by the other precipitation that was conducted with 7 M C_2_H_7_NO_2_ (Merck Darmstadt, Germany) for 2 h at −20 °C, and samples were centrifugated for 20 min at 17,000× *g* 4 °C. Two consecutive washes were carried out with 80% EtOH before RNA was eluted in 100 µL of RNAse-free water to continue with RNA Cleanup Protocol from RNeasy Mini Kit (Qiagen, Valencia, California, USA), according to the manufacturer’s instructions, before storage at −80 °C. RNA was quantified and assessed for quality using a Qubit 2.0 fluorometer RNA Assay Kit (Invitrogen Inc., Carlsbad, CA, USA) and an Epoch Take3 microplate spectrophotometer (BioTek Instruments Inc., Winooski, VT, USA), respectively. Additionally, RNA integrity was confirmed by denaturing electrophoresis, as previously described in [57]. To eliminate potential genomic DNA contamination, all RNA samples were treated with a DNase utilizing TURBO DNA-free Kit (Ambion™, Invitrogen Inc., Carlsbad, CA, USA) according to the manufacturer’s recommendations, and cDNA synthesis was conducted using a SuperScript™ First-Strand Synthesis System for RT-PCR (Invitrogen, Inc. USA) following the manufacturer’s instructions.

### 4.3. qRT-PCR and Selection of Candidate Genes

Analyses of relative transcript levels were performed, as described in [57], with some adaptations: three biological replicates (with three technical replicates) were independently prepared for each point sampled up to 10 µL final volume reaction, using KAPA SYBR FAST qPCR Kit (Life Technologies, Waltham, MA, USA) as 2X MasterMix for each RT-qPCR reaction with a 1:20 dilution of cDNA as template, including 2.5 pmol of forward and reverse primers. The *VvEFIα* gene (GSVIVG01025147001; translational elongation factor EF1A) was selected as the normalizer to the transcript levels of genes of interest, between the genes *VvMVK* (GSVIVG01000037001; mevalonate kinase) and *VvUBQ* (GSVIVG01008590001; ubiquitin family protein). PCR efficiencies were determined for each sample using LinRegPCR software v7.5 [58], to analyze the data with the 2^−ΔΔCT^ method with an efficiency correction [59]. Genes were selected from an RNA-Seq experiment derived from cv. Thompson Seedless samples collected at flowering and fruit set stages analyzed in [29]. The normalized expression data (FPKM) of early stages (BBCH65 and BBCH71) were used to select candidate genes to be experimentally validated by RT-qPCR. Thus, as selection criteria, genes exhibiting differences in gene expression over 50 FPKM among flowering and fruit-set stages were chosen, and the functional annotation of genes was also considered. Gene-specific primers were designed by Primer Premier 6 (Premier Biosoft International, Palo Alto, CA, USA) to target cell wall-related and cell cycle-related genes, utilizing the mRNA sequences downloaded from www.phytozome.com, accessed on 20 September 2021. The list of primers and its sequence is detailed in Table S1.

### 4.4. Optical Microscopy and Immunofluorescence

#### 4.4.1. Inflorescence Fixation and Section

Nine fixed individual flowers from inflorescences at the BBCH65 stage, each from a different plant (*n* = 3), were detached from the calyptra with a scalpel and straightaway dehydrated in six-step consecutive cold ethanol solutions with increasing concentrations (10%, 30%, 50%, 70%, 95%, and 100%; *v*/*v*), according to [27]. The samples were immediately saturated with xylol in rising concentrations of xylol/ethanol solutions (20/80, 40/60, 60/40, 80/20, and 100% xylol; *v*/*v*), and then embedded in paraffin by successively transferring them into paraffin/xylol solutions (20/80, 50/50, 75/25, and 100%; *v*/*v*) at 60 °C, to let them polymerize in stainless steel molds at room temperature. Subsequently, ovary transversal microsections (5 µm) were cut using a microtome (Leica RM 2125 RT, Leica Biosystems, Nussloch, Germany) and adhered to the slides; then, paraffin was removed by two washings over 10 min with 100% xylol. Then, the sample sections were rehydrated through a serial of ethanol/PBS (*v*/*v*; phosphate-buffered saline) solution with decreasing concentrations to finalize in a PBS 1X 100% washing. In the case of samples immunolabeled with 2F4 monoclonal antibody, ethanol/(T/Ca/S) buffer (Tris-HCl 20 mM pH 8.2, CaCl_2_ 0.5 mM, NaCl 150 mM) was used.

#### 4.4.2. Light Microscopy—Histochemical Staining

After deparaffination, the samples were treated according to [17], with slight modifications. Briefly, the slides were immersed in Safranin for one hour, then were gently washed twice with distilled water. The stained samples were immediately dehydrated through washes with increasing ethanol/water solutions (50%, 70%, 95%, and 100%; *v*/*v*), submerged in Fast Green for two minutes, and finally washed with eugenol for ten minutes to remove the excess staining. The stained sections were viewed under an optical microscope Leica DM500, and images were acquired with the attached camera Leica ICC50W (Leica Macrosystems, Wetzlar, Germany). Image processing was performed with Fiji [60]. Photographs of six ovaries per inflorescence were analyzed to determine the ovary diameter, number of cells of the external epidermis, and the outer mesocarp from CPPU-treated and control plants. To estimate the average ovary diameter, five measurements per ovary were made by drawing one line perpendicular to the septum, two lines parallel to the septum, and two lines through the major diagonals (Figure 2). To quantify the number of cells in the external epidermis, the cells were counted on 200 µm along a drawn line, at six different and representative arcs per ovary (Figure 2). Outer and inner mesocarps (OM and IM) were delimited by the presence of vascular bundles [4], and six elliptical areas of 5000 µm^2^ were established per ovary to count the number of cells in the outer mesocarp (OM) (Figure 2). Counts were carried out within the defined surface or distance, considering any cell for which more than 50% of its size was included within the lines.

#### 4.4.3. Confocal Laser Scanning Microscopy—Immunofluorescence

Deparaffinized samples were treated according to [27], with some modifications. After rehydration, ovaries were incubated with 0.4% (*v*/*v*) Triton X-100 in 1X PBS for two hours and blocked using 3% (*w*/*v*) milk protein in 1X PBS at room temperature. The monoclonal antibodies used were 2F4 and LM15, purchased from PlantProbes (Leeds, UK; www.plantprobes.net, accessed on 10 September 2021), in 1:50 dilution; secondary antibodies were anti-mouse IgG when 2F4 monoclonal was applied, or anti-rat IgG in the LM15 monoclonal antibody case, both conjugated to Alexa 488 (Thermo Fisher Scientific, Rockford, IL, USA) at a concentration of 1:500 diluted in T/Ca/S buffer (Tris-HCl 20 mM pH 8.2, CaCl_2_ 0.5 mM, NaCl 150 mM) or 1X PBS, respectively. To visualize the cell wall, Calcofluor White M2R (Sigma-Aldrich, St. Louis, MO, USA) was used for counterstaining at a concentration of 50 mg L^−1^ diluted in buffer (1X PBS or T/Ca/S). All the slides were mounted with Neo-Mount^®^ (Sigma-Aldrich, St. Louis, MO, USA). Confocal images were obtained with a Leica LSI confocal microscope (Leica Microsystems, Wetzlar, Germany) using an optical zoom of 3.6 and a digital zoom factor of 1.0. Images were processed using the software Leica Application Suite X (LASX) version 3.4.2 from Leica Microsystems (Wetzlar, Germany).

#### 4.4.4. Quantification of Immunofluorescence Signal

Calcofluor White signal from the ovary wall was estimated using Fiji, according to [61]. The procedure was as follows: each image was converted to an eight-bit image, and the threshold parameters were set as the same for all the samples. Immediately, the outside area (external epidermis) was delimited with the ‘area selection’ tool, and the identical mode was performed to measure the internal area of the ovary (internal epidermis delimitation). Both areas were determined with the ‘measure’ tool from the ‘analyze’ menu, selecting ‘area’, ‘Min and Max gray value’, ‘Integrated density’, and ‘Mean gray value’. To settle the corrected total fluorescence (CTF), Equation (1) was used:

CTF = Integrated density − (area selected × mean fluorescence of background readings)(1)

where mean fluorescence backgrounds correspond to areas without Calcofluor White signal. All the measures were corrected to the total area of the ovary wall of each sample.

### 4.5. Analysis of Cell Wall Monosaccharide Composition

Epicarp and mesocarp from berries at harvest were separated with a scalpel under frozen conditions to avoid thawing and immediately ground in liquid nitrogen with a mortar. Then, AIR (alcohol-insoluble residues) extraction, hydrolysis, and high-performance anion-exchange chromatography with pulsed amperometric detection (HPAEC-PAD) conditions were carried out according to [62], with slight modifications. First, the frozen ground samples (3 g) were incubated in agitation for two hours, with 40 mL of 80% (*v*/*v*) ethanol at room temperature, followed by centrifugation at 6000× *g* 15 min and the supernatant was discarded; this procedure was repeated three times. The final step carried out was washing with 100% acetone and centrifugation to obtain the solid residue while drying overnight at room temperature under a fume hood. The AIR (2 mg), cell wall-enriched fraction, was hydrolyzed for 1 h with 400 µL of 2 M trifluoroacetic acid (TFA) at 121 °C. Then, TFA was evaporated at 65 °C for 30 min using nitrogen, and the resultant pellets were washed twice with 300 µL of 100% isopropanol, followed by Speed-Vac evaporation (Eppendorf, Hamburg, Germany). The hydrolyzed samples were suspended in 1 mL of Milli-Q water, sonicated, and filtered through a 0.45 µm pore-size syringe filter, and injected into an HPAEC-PAD (Dionex DX-600 Ion Chromatography System, Dionex Corp., Sunnyvale, CA, USA). For the analysis, there were two CarboPac PA1 (4 mm × 250 mm) analytical columns connected in series, including a CarboPac PA1 (4 mm × 50 mm) guard column to quantify neutral sugars (fucose, rhamnose, arabinose, galactose, glucose, xylose, and mannose), and acidic sugars (galacturonic acid and glucuronic acid). The separation was performed at 30 °C with a flow rate of 1 mL min^−1^, with elution by isocratic gradients of 20 mM NaOH for 20 min, followed by 150 mM sodium acetate in 100 mM NaOH for 15 min. A final washing step was performed with 200 mM NaOH for 10 min. Sugar content for each sample was determined using a standard curve and expressed as mg g^−1^ AIR, and allose was used as an internal control. After every run, the column was equilibrated in 20 mM NaOH for 10 min.

### 4.6. Calcium Quantification

Inductively coupled plasma optical emission spectrometry (ICP-OES) was used to analyze the total content of calcium of the AIR from berries at harvest, as detailed by [2], using a Thermo Scientific iCAP 6000 Series ICP Emission Spectrometer (Thermo Scientific, Tewksbury, MA, USA) to measure the samples at 393.3 nm. Results were expressed as mg of calcium per kg^−1^ AIR.

### 4.7. Harvest Berry Phenotypic Analysis

Ten berries per bunch were phenotyped to measure berry weight, diameter, total soluble solids (TSS, %), titratable acidity (TA, % citric acid), and color. Additionally, texture profile analysis was performed with a TA.XT plus Texture Analyzer (Stable Micro Systems Ltd., Godalming, UK), according to [27], with some modifications. Briefly, each berry was compressed in the equatorial diameter by SMS P/35 flat probe (2.5 cm × 2.5 cm: Stable Micro Systems), under a deformation of the berry of 20% with a waiting time between the two bites of 2 s using 1 mm s^−1^ as speed test.

### 4.8. Statistical Analysis

Data were analyzed using the R environment (RStudio Inc., Boston, MA, USA, v 1.2.1335), the specific package ‘agricolae’, in order to examine the treatment effects by Student’s unpaired, two-tailed *t*-test (*p* = 0.05).

## 5. Conclusions

Taken together, our results indicate that CPPU treatment prior to anthesis produces an effect at the cell wall level, as evidenced by differences at the morphological level of ovaries at anthesis and, consequently, in berry firmness at harvest. Analysis of the relative expression of cell cycle and cell wall-related genes of CPPU-treated inflorescences showed an increased expression due to cytokinin treatments, indicating that increased cell division caused by cytokinin applications involves cell wall restructuring. These findings correlated with larger ovaries at anthesis in treated plants, accompanied by an increased cell number in the outer mesocarp. Cell wall restructuring evidenced by the increased HG epitope signal for the outer mesocarp in CPPU-treated ovary plants and increased signal for egg-box structures in inner epidermal cells suggests structure modifications at a very early stage of development that anticipate a phenotype of increased firmness at harvest. These results evidenced harder berries with a higher calcium content in the cell wall, which was obtained in CPPU plants when compared to control plants. Our work presents new knowledge on a CPPU pre-anthesis treatment in table grapes and its effects on berry development, correlating cell wall rearrangement at a very early stage of inflorescence and ovary development with higher firmness at harvest.

## Data Availability

Not applicable.

## Supplementary Materials

The following are available online at https://www.mdpi.com/article/10.3390/plants10122642/s1. Table S1: Primer sequences of genes used for RT-qPCR analysis.
