# Supplementary material for: Pre-Anthesis Cytokinin Applications Increase Table Grape Berry Firmness by Modulating Cell Wall Polysaccharides
Source: Plants (Basel). 2021 Dec 1;10(12):2642. doi: 10.3390/plants10122642 (PMC8708260; doi:10.3390/plants10122642)
Supplement: Supplementary file 1 [file plants-10-02642-s001.zip › plants-1420193-supplementary.pdf]

Table S1. Primer sequences of genes used for RT-qPCR analysis.

Gene Name	ID Gene (Genoscope.12X)	Sequence
VvCDKF-4	GSVIVT01022771001	F 5'-GGAAGTGTTTGGCGAGCAAT-3'<br>R 3'-CCCTGAGCTTCACAATGTTTGG-5'
VvCYC3-1	GSVIVT01011079001	F 5'- GTTATAGGAACCCCTCGGGTTT-3'<br>R 3'-TGGGCTACAGCCTGAGTACTT-5'
VvPME	GSVIVG01028041001	F 5'- CCAACTCCAGCACTACCAG-3'<br>R 3'-TCGGTTGCCCTTGATGTAAG-5'
VvPG	GSVIVG01026985001	F 5'- TCAACTCGCCTTCATGGAAC-3'<br>R 3'-CAATGTAGCAGTCTTCAATCCTTG-5'
VvEFIα	GSVIVG01025147001	F 5'-GAACTGGGTGCTTGATAGGC-3'<br>R 3'-AACCAAAATATCCGGAGTAAAAGA-5'
VvMVK	GSVIVG01000037001	F 5'- ATTGCCAACCCTGCTATCAGGAAC-3'<br>R 3'- CGCCGATTCCAGCGATAAGACATT-5'
VvUBQ	GSVIVG01008590001	F 5'- GAACTTGCAGGAGGGGTTCTTGTA- 3'<br>R 3'- GAGCAGACAACTTTCTTGGGTGACT- 5'
